# Cognitive and psychomotor responses to high-altitude exposure in sea level and high-altitude residents of Ecuador

**DOI:** 10.1186/s40101-014-0039-x

**Published:** 2015-02-04

**Authors:** John E Davis, Dale R Wagner, Nathan Garvin, David Moilanen, Jessica Thorington, Cory Schall

**Affiliations:** Department of Integrative Physiology and Health Science, Alma College, 614 Superior Street, Alma, MI 48801 USA; Human Movement Science Program, Utah State University, 7000 Old Main Hill, Logan, UT 84322 USA

**Keywords:** Altitude, Adaptation, High-altitude natives, Cognitive function

## Abstract

**Background:**

High-altitude inhabitants have cardiovascular and respiratory adaptations that are advantageous for high-altitude living, but they may have impaired cognitive function. This study evaluated the influence of altitude of residence on cognitive and psychomotor function upon acute exposure to very high altitude.

**Findings:**

Ecuadorians (31 residing at 0–1,500 m [LOW], 78 from 1,501–3,000 m [MOD], and 23 living >3,000 m [HIGH]) were tested upon their arrival to a hut at 4,860 m on Mount Chimborazo. Cognitive/psychomotor measurements included a go-no-go test (responding to a non-visual stimulus), a verbal fluency test (verbalizing a series of words specific to a particular category), and a hand movement test (rapidly repeating a series of hand positions). Mean differences between the three altitude groups on these cognitive/psychomotor tests were evaluated with one-way ANOVA. There were no significant differences (*p* = 0.168) between LOW, MOD, and HIGH for the verbal fluency test. However, the go-no-go test was significantly lower (*p* < 0.001) in the HIGH group (8.8 ± 1.40 correct responses) than the LOW (9.8 ± 0.61) or MOD (9.8 ± 0.55) groups, and both MOD (97.9 ± 31.2) and HIGH (83.5 ± 26.7) groups completed fewer correct hand movements than the LOW (136.6 ± 37.9) subjects (*p* < 0.001).

**Conclusions:**

Based on this field study, high-altitude residents appear to have some impaired cognitive function suggesting the possibility of maladaptation to long-term exposure to hypobaric hypoxia.

## Background

Acute decrements in cognitive and psychomotor performance at high altitude are common, but not as well understood as the physiological adaptations. In a review, Petrassi et al. [1] noted that there is considerable individual variability in the extent of cognitive impairment at altitude, but there is strong evidence for some learning impairment at altitudes as low as 8,000 ft (2,438 m). A variety of different types of cognitive assessments have been used at altitude, including number- and letter-sequence recognition, word association/generation tasks, and short- and long-term memory [2,3]. Most of these studies indicate that the cognitive impairments are directly related to the magnitude of hypoxia.

Several studies have also reported alterations in psychomotor function with altitude. Berry et al. [4] and Townes et al. [5] found finger-tapping tasks were significantly impaired after exposure to high altitude. Audition and early visual processing are also affected by hypoxia especially where speed of processing is important [6,7].

While a number of studies have looked at the acute responses to high altitude, very few studies have considered how cognitive function is affected by long-term exposure and adaptation. In a study comparing low- and high-altitude residents, high-altitude residents had lower accuracy in a verbal-working memory task, longer reaction times in spatial- and verbal-working memory tasks, and lower activation in the frontal and occipital cortices [8]. Niermeyer et al. [9] found cognitive impairments in high-altitude natives.

There is some speculation that these deficits could be due to early hypoxia-ischemia, nutritional deficits, and/or socioeconomic considerations. Several studies have found lower oxygen saturation in infants in communities above 3,000 m [10,11]. This early exposure to hypoxia might impair cognitive development. There has also been speculation that baby-rearing cultural practices [12] and nutritional deficiencies such as iron-deficiency anemia [13] might contribute to cognitive deficits in children living at high altitude [13]. However, confounding effects like genetic differences have made interpretation of the data difficult.

Therefore, the purpose of this study was to evaluate the influence of altitude of residence on cognitive function and psychomotor responses upon acute exposure to very high altitude. To our knowledge, this is the first study to compare altitude of residence and eliminate the influence of using different ethnic populations.

## Methods

### Study design

This was a comparative study using a convenience sample.

### Setting and participants

Data collection occurred inside a hut at 4,860 m that can be reached by vehicle in a popular national landmark area of Ecuador. Ecuadorians who arrived at the hut during the data collection period were informed of the study and invited to participate. Following institutional ethical board approval at Alma College, volunteers gave their written consent to participate in the study in accordance with the Declaration of Helsinki. Volunteers read and signed a written informed consent that was available in Spanish. Special care was taken to ensure they understood all of the procedures. All participants were given a medical/health history and excluded if they had any disease that would significantly impact the testing procedure.

### Procedures

Data collection occurred over a period of 2 weeks during the month of June in 2009, 2010, and 2011. Participants drove to the hut from their altitude of residence within 24–48 h. No subjects were using supplemental oxygen or Diamox for acute mountain sickness. Upon completion of the informed consent, three cognitive tests were administered to each subject. The tests comprise the Frontal/Subcortical Assessment Battery [14]. This brief test battery of cognitive and motor functions provides a valid assessment of mild subcortical dementia [14] and has been used previously at high altitude (Marco-Duenas, unpublished). All tests were given within an hour after the subjects reached the hut, thereby emphasizing the acute response to high altitude by minimizing adaptation effects. Two trained investigators administered all of the tests. Each participant demonstrated that they understood the task by performing an example; however, there was no full practice period to minimize the learning effect.

The three-item test battery included a test of verbal fluency during which subjects were asked to name three animals, then three vegetables, then three different animals, and so on for 60 s. The second test was a hand movement test during which subjects produced sequential hand gestures (fist, palm up, palm to the side) as quickly as possible for 60 s. The final test was the go-no-go test. For this test, the subject tapped the table twice in response to a single tap by the examiner, who was out of the subject’s view, and did not tap the table at all in response to two taps by the examiner. The number of correct responses was recorded for each item in the test battery.

### Statistical analyses

Data were separated into three groups based on the altitude of residence of the participants: LOW (0–1,500 m), MOD (1,501–3,000 m), and HIGH (>3,000 m). Analysis of variance (ANOVA) with Tukey’s *Post Hoc* test was used to identify statistically significant mean differences between groups. Statistical significance was set at *p* < 0.05. All statistical tests were done with SPSS (version 22, IBM, Armonk, NY). Means ± 1 SD are plotted in all figures.

## Results and discussion

**Figure 1:**
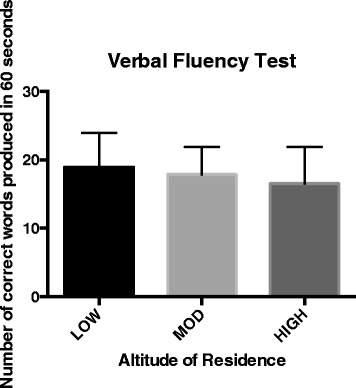
**Number of correct words produced in 60 s for the LOW, MOD, and HIGH groups.** There were no significant differences between residence altitudes (*p* = 0.168).

A total of 132 Ecuadorians (31 LOW, 78 MOD, 23 HIGH) completed the study. There was no significant difference in age, body mass, height, arterial oxygen saturation (SaO2), or a self-assessment of acute mountain sickness (LLSS) between groups (Table 1). For the verbal fluency test, HIGH produced the fewest number of words (Figure 1); however, there were no significant differences (*F*_2,130_ = 1.807, *p* = 0.168) between groups. Virués-Ortega and colleagues [15] highlighted numerous examples of impaired verbal fluency as a result of high-altitude exposure in their review of neuropsychological functioning associated with this environment. However, the majority of these studies involved mountaineers going to extreme altitudes. Other researchers have shown that verbal impairment due to high altitude occurs only at 6,000 m or higher [16] and is only temporary, returning to baseline upon return to sea level [17].

**Table 1:** **Descriptive data of the sample (mean ± SD)**

**Altitude (m)**	**Age (year)**	**Body mass (kg)**	**Height (m)**	**SaO2 (%)**	**LLSS**	**Gender**
Low (0–1,500)	40.3 ± 14.2	68.5 ± 10.5	1.68 ± 0.35	72.3 ± 11.5	1.6 ± 1.0	24 m, 7 f = 31
Mod (1,501–3,000)	37.0 ± 12.8	72.4 ± 15.5	1.67 ± 0.22	78.7 ± 8.0	1.7 ± 0.9	46 m, 32 f = 78
High (>3,000)	37.5 ± 17.7	71.2 ± 12.3	1.70 ± 0.48	82.8 ± 9.8	1.6 ± 1.2	15 m, 8 f = 23

*SaO2* arterial oxygen saturation, *LLSS* Lake Louise Self-Score for acute mountain sickness, *m* male, *f* female.

**Figure 2:**
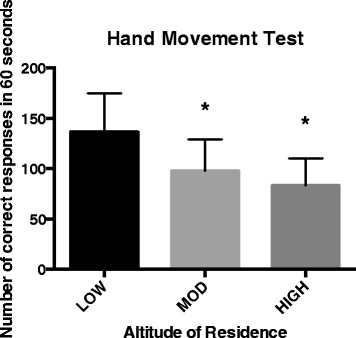
**Number of correct repetitions in 60 s for the hand movement test for the LOW, MOD, and HIGH groups.** Both the MOD and HIGH groups completed fewer correct hand movements than the LOW subjects (*p < 0.001), although the difference between MOD and HIGH was not significant (*p* = 0.148).

The same trend of HIGH and MOD having fewer correct responses existed for the hand movement sequence (Figure 2). This time, the ANOVA was significant (*F*_2,130_ = 22.382, *p* < 0.001) with an effect size of *η*^2^ = 0.26. Both the MOD and HIGH groups completed fewer correct hand movements than the LOW subjects, although the difference between MOD and HIGH was not significant (*p* = 0.148). Our finding of impaired motor function supports the work of Berry et al. [4] who reported reduced speed and precision of finger tapping at high altitude, and our results suggest that this impairment is independent of ethnicity. As to whether this is a direct response of living in a hypoxic environment or some other factor has yet to be determined. Paola et al. [18] found that extremely high-altitude exposures may cause subtle reductions in white and grey matter in the brain regions involved in motor function. However, again, these data are specific to mountaineers going to extreme altitudes over several weeks and cannot necessarily be applied to individuals residing at 3,000 m.

**Figure 3:**
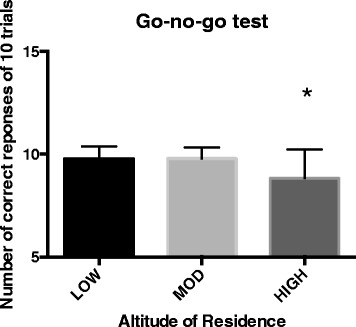
**Number of correct responses of 10 trials of the go-no-go test for the LOW, MOD, and HIGH groups.** The asterisk indicates a significant difference (*p* < 0.001) between LOW and MOD verses HIGH groups.

The correct number of responses to the go-no-go non-visual stimulus test was significantly less in HIGH compared to LOW or MOD (Figure 3; *F*_2,129_ = 14.282, *p* < 0.001) with an effect size of *η*^2^ = 0.18. This test served as a measure of a participant’s capacity for sustained attention and response control. The lower score for the HIGH group could be attributed to the lack of the need to respond to environmental cues, for example traffic and phones, or a direct influence of the hypoxia.

Given that the present study focused on Andean high-altitude natives, it is possible that other high-altitude natives might respond differently in light of recent studies indicating quite different evolutionary strategies to high-altitude adaptation in Tibetans verses Andeans [19]. Another limitation was that a convenience sample was tested, and there were more than twice as many MOD participants than LOW or HIGH residents. However, one could argue that this is simply a reflection of the demographics of the region as many of the surrounding cities and villages are at altitudes between 1,500 and 3,000 m. A further limitation with a convenience sample is that no baseline measurements were made at the participants’ residences; thus, no change scores between 4,860 m and their altitude of residence are available. It is logical to assume that an acute hypoxic exposure would have less of a detrimental effect on high-altitude residents who are somewhat acclimatized than on sea-level residents; however, we cannot definitively state that the altitude of residence was more influential than the acute change in altitude for the differences in scores between groups. We can only state that we observed some diminished cognitive and psychomotor scores for the higher-altitude residents compared to their lower-altitude compatriots when tested at 4,860 m, but based solely on simple field tests without baseline data, it is not possible to determine the etiology of these differences. More research is needed to determine if these differences are due to cognitive impairments as a result of chronic hypoxia, cultural or sociological differences, educational status, nutritional deficiencies, a combination of these, or other factors.

## Conclusions

High-altitude residents appear to have some indication of impaired cognitive function without symptoms of neurological disease, suggesting the possibility that long-term exposure to hypoxia might result in a maladaptation to long-term high-altitude living.
